# Assessment of the Angiogenic Potential of 2-Deoxy-D-Ribose Using a Novel *in vitro* 3D Dynamic Model in Comparison With Established *in vitro* Assays

**DOI:** 10.3389/fbioe.2019.00451

**Published:** 2020-01-17

**Authors:** Serkan Dikici, Betül Aldemir Dikici, Shirin Issa Bhaloo, Mercedes Balcells, Elazer R. Edelman, Sheila MacNeil, Gwendolen C. Reilly, Colin Sherborne, Frederik Claeyssens

**Affiliations:** ^1^Department of Materials Science and Engineering, Kroto Research Institute, University of Sheffield, Sheffield, United Kingdom; ^2^Institute for Medical Engineering and Science, Massachusetts Institute of Technology, Cambridge, MA, United States; ^3^Department of Materials Science and Engineering, INSIGNEO Institute for In Silico Medicine, University of Sheffield, Sheffield, United Kingdom; ^4^Bioengineering Department, Institut Quimic de Sarria, Ramon Llull University, Barcelona, Spain; ^5^Division of Cardiovascular Medicine, Department of Medicine, Harvard Medical School, Brigham and Women's Hospital, Boston, MA, United States

**Keywords:** angiogenesis, vascular endothelial growth factor (VEGF), 2-deoxy-D-ribose (2dDR), thymidine phosphorylase (TP), angiogenesis assays, shear stress, emulsion templating, PolyHIPE

## Abstract

Angiogenesis is a highly ordered physiological process regulated by the interaction of endothelial cells with an extensive variety of growth factors, extracellular matrix components and mechanical stimuli. One of the most important challenges in tissue engineering is the rapid neovascularization of constructs to ensure their survival after transplantation. To achieve this, the use of pro-angiogenic agents is a widely accepted approach. The study of angiogenesis has gained momentum over the last two decades. Although there are various *in vitro, ex vivo*, and *in vivo* angiogenesis models that enable testing of newly discovered pro-angiogenic agents, the problem with researching angiogenesis is the choice of the most appropriate assay. *In vivo* assays are the most representative and reliable models, but they are expensive, time-consuming and can cause ethical concerns whereas *in vitro* assays are relatively inexpensive, practical, and reproducible, but they are usually lack of enabling the study of more than one aspect of angiogenesis, and they do not fully represent the complexity of physiological angiogenesis. Therefore, there is a need for the development of an angiogenesis model that allows the study of angiogenesis under physiologically more relevant, dynamic conditions without causing ethical concerns. Accordingly, in this study, we developed 3D *in vitro* dynamic angiogenesis model, and we tested the angiogenic potential of 2-deoxy-D-ribose (2dDR) in comparison with vascular endothelial growth factor (VEGF) using newly developed *in vitro* 3D dynamic model and well-established *in vitro* models. Our results obtained using conventional *in vitro* assays demonstrated that 2dDR promoted proliferation, migration and tube formation of human aortic endothelial cells (HAECs) in a dose-dependent manner. Then, the angiogenic activity of 2dDR was further assessed using the newly developed 3D *in vitro* model, which enabled the monitoring of cell proliferation and infiltration simultaneously under dynamic conditions. Our results showed that the administration of 2dDR and VEGF significantly enhanced the outgrowth of HAECs and the cellular density under either static or dynamic conditions.

##  Introduction

Angiogenesis is a complex process driven by the interactions of endothelial cells (ECs) with growth factors, extracellular matrix, and mechanical stimuli (Risau, 1997). Delayed angiogenesis of tissue-engineered (TE) constructs is a major challenge for their translation to the clinic (Novosel et al., 2011). One approach is the use of pro-angiogenic agents to circumvent slow neovascularization. Thus, researchers have been exploring pro-angiogenic agents to ensure rapid neovascularization in tissue engineering constructs using well-established *in vitro* and *in vivo* models.

Vascular endothelial growth factor (VEGF) is a well-known stimulator of angiogenesis and recognized as the most effective pro-angiogenic factor both *in vitro* and *in vivo* (Ferrara, 2009). However, several studies showed that the administration of VEGF in an uncontrolled manner might cause excessively leaky (Yancopoulos et al., 2000), permeable (Cao et al., 2004), and haemorrhagic (Cheng et al., 1997) vessels such as those that are found in the process of tumor angiogenesis (Oka et al., 2007). Controlled and sustained release of VEGF may help to regulate the delivery rate of VEGF and circumvent these problems by creating mature, more durable and stable vessels (Chen et al., 2007; Ehrbar et al., 2008; Le et al., 2009; Formiga et al., 2010). However, it is challenging to fabricate systems that bind and deliver VEGF in a controlled manner because most process developed to date are time-consuming, multi-step and expensive processes. In general, translation of the exogenous use of VEGF into the clinic is difficult (Ferrara and Alitalo, 1999). Thus, exploring alternatives to VEGF is crucial for ensuring rapid and safe neovascularization in TE constructs.

Thymidine phosphorylase (TP), an enzyme which catalyzes the reaction of thymidine into thymine, has been identified as platelet-derived EC growth factor (PD-ECGF) (Friedkin and Roberts, 1954; Furukawa et al., 1992). Although its molecular mechanism is still unclear, the enzymatic activity of TP has previously been reported to be angiogenic *in vivo* (Ishikawa et al., 1989; Miyadera et al., 1995; Moghaddam et al., 1995). Therefore, TP-dependant angiogenesis studies focus on 2-deoxy-D-ribose (2dDR), one of the degradation products of thymidine. 2dDR has previously demonstrated to have chemotactic and angiogenic activity *in vivo* (Haraguchi et al., 1994; Matsushita et al., 1999). Similarly, our group recently demonstrated the angiogenic potential of 2-deoxy-D-ribose (2dDR) using an *ex-ovo* CAM assay (Dikici et al., 2019b) and a diabetic rat model (Azam et al., 2019). Several groups evaluated the angiogenic activity of 2dDR *in vitro* but only using a narrow range of concentrations. Thus, the dose-dependent response of 2dDR still needs to be investigated *in vitro*.

Angiogenesis models are important tools to study the angiogenic activity of agents, biomechanical stimulus and cells (Staton et al., 2009). Current angiogenesis assays can be divided into three categories: *in vitro* assays which focus on evaluating proliferation, migration, and tube formation capabilities of ECs, *ex vivo assays* such as rat aortic ring assay, chick aortic arch assay, animal retina model, and *in vivo* assays which are chick chorioallantoic membrane (CAM), zebrafish, corneal angiogenesis, xenograft, and Matrigel® plug assays (Stryker et al., 2019).

Although *in vivo* assays are the most representative and reliable models for the evaluation of angiogenesis, they are also expensive, technically difficult, time-consuming, and ethically questionable (Staton et al., 2004). On the other hand, *in vitro* angiogenesis assays are inexpensive, quick, technically simple, and reproducible, but they are usually based on evaluating only one aspect of angiogenesis (for example, proliferation, migration, or differentiation), and they may produce false results due to the non-specific reaction of cells (Bahramsoltani et al., 2009). Moreover, most of the *in vitro* angiogenesis assays are limited to static, two-dimensional (2D) cell culture systems where culturing cells on stiff and flat substrates is a simplified method and does not represent the dynamic and highly complex tissue systems (Hutmacher, 2010; Ravi et al., 2015). 2D culture of cells distorts cell-cell and cell-matrix interactions which affects cell proliferation, migration and differentiation (Cukierman et al., 2001; Pampaloni et al., 2007), whereas 3D *in vitro* models better represent the *in vivo* situation in a cost-effective way and with no ethical concerns (Ravi et al., 2015). To date, none of the studies demonstrated an *in vitro* model which allows researchers to evaluate angiogenesis assessing both proliferation and migration of ECs in a 3D dynamic environment.

In this study, we evaluated the angiogenic potential of 2dDR using established *in vitro* angiogenesis assays, and we assessed the potential of the newly developed tubular 3D dynamic model to be used in the evaluation of two phases of angiogenesis together; cell proliferation and migration in a dynamic environment. The 3D system was designed in a bilayer tubular design. The nanofibrous structure of the inner tube mimics the basement membrane and provides a suitable environment for EC attachment and formation of an endothelial monolayer whereas the highly porous and interconnected outer polymerised high internal phase emulsion (PolyHIPE) tube enables cell infiltration and migration through the pores.

## Materials

Pentaerythritol (98%), ε-caprolactone, tin (II) 2-ethylhexanoate, triethylamine (TEA), methacrylic anhydride (MAAn), photoinitiator (PI) (2,4,6-Trimethylbenzoyl Phosphine Oxide/2-Hydroxy-2-Methylpropiophenone blend), trypsin, paraformaldehyde (PFA), resazurin sodium salt, ethanol, hematoxylin solution, eosin Y solution, trypan blue, Triton X-100, Polydimethylsiloxane (PDMS, silicone), vascular endothelial growth factor (human), 2-deoxy-D-ribose (2dDR), glutaraldehyde (GA), goat serum, fetal calf serum (FCS) and crystal violet were purchased from Sigma Aldrich (St. Louis, Missouri, USA). Chloroform, industrial methylated spirit (IMS), toluene, dichloromethane (DCM) and methanol were purchased from Fisher Scientific (Pittsburgh, PA, USA). The surfactant Hypermer B246-SO-M was received as a sample from Croda (Goole, UK). 4′,6-diamidino-2-phenylindole (DAPI) solution and Alexa Fluor 594 Phalloidin were purchased from ThermoFisher Scientific (San Jose, CA, USA). Optimum cutting temperature tissue freezing medium (OCT-TFM) was purchased from Leica Biosystems (Newcastle, UK). Collagenase A was purchased from Roche (Indianapolis, IN, USA). Human Aortic Endothelial Cells (HAECs) and EC Growth Medium (EC GM) were purchased from PromoCell (Heidelberg, Germany). Poly3-hydroxybutyrate-co-3-hydroxyvalerate (PHBV) was purchased from Goodfellow (London,UK). Alexa Fluor® 594 anti-human CD31 Antibody was purchased from Biolegend (San Diego, CA, USA). Cell culture inserts and Matrigel® were purchased from Corning (New York, NY, USA).

## Methods

### Assessment of the Angiogenic Potential of 2dDR Using Established *in vitro* Assays

#### Human Aortic Endothelial Cell (HAEC) Culture

HAECs removed from liquid nitrogen were immediately thawed at 37°C and transferred to a container with 10 mL of EC growth medium (GM). Cells were centrifuged at 1,000 rpm for 5 min, and the cell pellet was re-suspended in EC GM supplemented with a total of 5% fetal calf serum (FCS) and growth medium two kit supplements. The cell suspension was then transferred into 75 cm^2^ flasks and incubated at 37°C, 5% CO_2_ (Thermo Scientific, Massachusetts, USA). The culture media was replaced every 3 days until they reached ~80–90% confluency. HAECs were used between passages two and six.

#### Preparation of Pro-angiogenic Agents

Working solutions of all substances were prepared fresh at the beginning of each experiment. 10 mM 2dDR stock solution was prepared by dissolving in low serum (2% FCS) EC GM and filtered using a 0.2 μm syringe filter. 1, 10, 100 μM, and 1 mM concentrations of 2dDR were prepared by diluting the 10 mM 2dDR stock solution in low serum EC GM. 80 ng/mL VEGF solution was prepared in EC GM and used as a positive control. VEGF will be used to describe VEGF 80 ng/mL throughout the paper unless otherwise stated. Low serum EC GM was used as a control in the experiments.

#### Evaluating the Effect of 2dDR on the Proliferation of HAECs

##### Alamar blue metabolic activity assay

Alamar Blue Cell Viability Assay was performed to evaluate the effect of different concentrations of 2dDR on HAECs growth *in vitro*. The principle of this assay is the reduction of non-fluorescent resazurin to highly fluorescent resorufin by the metabolically active cells.

Once the HAECs reached ~80–90% confluency, they were trypsinized and seeded into 48-well plates with a seeding density of 1 × 10^4^ HAECs/cm^−1^. Alamar Blue Cell Viability Assay was performed at days 1, 4, and 7. Briefly, 1 mM of Alamar Blue stock solution was prepared by dissolving 0.025 g of resazurin sodium salt in 100 ml of deionised water and filter sterilized. 0.1 mM Alamar Blue working solution was prepared by 10× dilution of the 1 mM sterile Alamar Blue stock solution with EC GM. Growth medium was removed, and the cells were washed with PBS. One milliliter of Alamar Blue working solution was added to each well and incubated at 37°C for 4 h. After an incubation period of 4 h, 200 μL of the solution was transferred into 96-well plate, and the fluorescence readings were done at the excitation wavelength of 540 nm and an emission wavelength of 635 nm.

##### Fluorescent staining of cell nuclei and cytoskeleton

In order to visualize the cells after 7 days, fluorescent staining was performed by labeling F-actin and cell nuclei of HAECs. Cells were washed with PBS before (once) and after (three times) fixing them in 4% PFA for 15 min. 0.1% (v/v) Triton 100× (in PBS) was added on samples, and the samples were incubated for 20–30 min at room temperature (RT). After three times washing with PBS, Alexa Fluor 594 Phalloidin (1:40 diluted in PBS from stock solution) solution was added to cells in order to stain F-actin filaments of cells and incubated for 30 min at RT in the dark. Cells were then washed three times with PBS. Nuclei were stained with DAPI solution (1:1,000 diluted in PBS), which strongly binds the adenine-thymine rich regions of DNA. The DAPI solution was added to cells and incubated for 10–15 min at RT in the dark, and cells were then washed three times with PBS. Cells were then examined with a fluorescent microscope (Nikon Eclipse Ti, Tokyo, Japan).

##### CD31 staining

CD31 immunofluorescent staining was performed to evaluate the expression of CD31 of HAECs treated with different concentrations of 2dDR and VEGF. At day 7, cells were fixed with 4% PFA and washed with PBS. Cells were incubated with 5% goat serum to avoid non-specific binding. Alexa Fluor® 594 anti-human CD31 Antibody staining solution was prepared by the dilution of 1:50 in 5% goat serum. Cells were incubated with the antibody staining solution overnight at 4°C. HAECs were finally counterstained with DAPI (1:1,000 diluted in PBS) as described in section Fluorescent Staining of Cell Nuclei and Cytoskeleton after washing three times with PBS. CD31 expression was visualized under a fluorescent microscope after washing the cells with PBS.

#### Evaluating the Effect of 2dDR on Stimulating the Migratory Response of HAECs Using a Modified Boyden Chamber Migration Assay

The Boyden chamber assay was developed for analyzing leukocyte chemotaxis in the 1960s (Boyden, 1962). There are two compartments containing media, and these compartments are separated with a microporous membrane. Cells are seeded into the top compartment (a cell culture insert) while the chemoattractant is placed into the compartment below (well plate). Herein, we used a modified Boyden chamber assay to evaluate the migratory response of HAECs to 2dDR in comparison with VEGF.

Briefly, 800 μL of chemoattractant solutions and low serum EC GM (as control) were added to the 24-well plates (lower chamber), and 8 μm pore size cell culture inserts were placed onto the solutions carefully by avoiding bubble formation. HAECs were trypsinized, centrifuged, and 5 × 10^4^ HAECs resuspended in 300 μL of low serum EC GM were seeded into the cell culture inserts (upper chamber). After incubation for 4 h at 37°C, cells which were not migrated were removed from the upper surface of the cell culture insert by scraping with a cotton bud. Cell culture inserts were fixed with 4% PFA for 10 min, and the migrated HAECs were stained with 0.1% crystal violet (wt/vol in deionised water) solution for 10 min before washing three times with deionised water. Bright-field images were taken with the fluorescent microscope, and the migration was quantified with a multi-step image processing of the green channel images exported from microscope software (NIS-Elements, Tokyo, Japan).

Briefly, the green channel images were converted to binary images with ImageJ (Wayne Rasband, National Institutes of Health, USA), and the black pixels which are the cells) were counted from the histograms. Four areas of interest were chosen randomly from each image, and the mean number of migrated cells was taken for each group.

#### Evaluating the Effect of 2dDR on Tube Formation of HAECs Using Matrigel® Tube Formation Assay

*In vivo*, ECs are in direct contact with a basement membrane which is specific and biologically functional for enabling ECs to form tube structures (Kalluri, 2003). Matrigel® is the trade name of a gelatinous protein mixture produced from a hamster fibrosarcoma, which is rich in collagen IV, laminin, proteoglycans and growth factors. This biologically active protein mixture is used as an *in vitro* substitute for mimicking native basement membrane of ECs and promotes ECs to form tube-like capillary structures (Kleinman and Martin, 2005). The tube formation assay is widely used as a bioassay for one aspect of neovascularization *in vitro*.

We examined whether 2dDR stimulates the tube formation of HAECs using the Matrigel® tube formation assay. Briefly, working on the ice, 48-well plates were thickly coated with growth factor reduced Matrigel® by adding 120 μL of Matrigel® into each well. The plates were incubated at 37°C for 60 min to allow solidification of the Matrigel®. HAECs were trypsinized, centrifuged, and seeded on Matrigel®-coated plates at a density of 2.5 × 10^4^ cells/well and treated with 100 μM of 2dDR in comparison with VEGF and a non-supplemented control of EC GM. HAECs were incubated at 37°C for 18 h before fixing them in 2% PFA solution containing 0.1% GA for 15 min. Tube formation was quantified using Angiogenesis Analyzer plugin of ImageJ (Brown et al., 2016).

### Design, Manufacturing, and Characterization of the 3D Dynamic Model

The 3D system was designed to be two-layers; the inner tube of nanofibers to serve as a suitable environment for HAECs to attach, proliferate and form a monolayer to represent an endothelium, and the outer tube of the highly porous and interconnected environment to enable proliferation and migration of HAECs from the formed endothelial monolayer. 3D bilayer tubes used in this study were manufactured combining two manufacturing methods; emulsion templating and electrospinning, and they were characterized using scanning electron microscopy.

#### Manufacturing of the Outer Tube by Emulsion Templating

##### Synthesis of polycaprolactone (PCL) methacrylate

The PCL used in this study is 4-arm PCL methacrylate (4PCLMA) which we have previously reported the detailed synthesis (Aldemir Dikici et al., 2019b). Throughout the paper, the term ‘PCL polymerised high internal phase emulsion (PolyHIPE)' will be used to describe 4PCLMA PolyHIPE, unless otherwise stated.

Briefly, pentaerythritol (12 g, 0.088 mol) and ε-caprolactone (80.49 g, 0.353 mol) were mixed in a round flask at 160°C while stirring continuously at 200 rpm. When pentaerythritol was dissolved, tin (II) 2- ethylhexanoate (as a catalyst) was added, and the system was left for reaction overnight with stirring. Then, stirring was stopped, and the system was removed from the oil bath to cool down in the ambient atmosphere. Hydroxyl-terminated 4-arm PCL was obtained, and to methacrylate functionalise, it was dissolved in 300 mL of DCM, and then TEA (52.65 g, 0.52 mol) was added. The flask was placed in an ice bath. MAAn (80.22 g, 0.52 mol) was dissolved in 100 mL DCM and transferred into a dropping funnel. When the addition of MAAn was completed, the ice bath was removed, and the system was kept at RT overnight with stirring at 375 rpm. To remove the TEA, MAAn and the salts formed, the methacrylated PCL was washed three times with HCl solution (1 M, 1,000 mL), separated using a separating funnel, and then washed three times with pure deionised water. Almost all solvent was evaporated using a rotary evaporator. PCL was then dissolved in methanol, then precipitated out of methanol by placing in −80°C freezer. Three methanol washes were used, and any remaining solvent was removed by using the rotary evaporator. 4PCLMA was stored in an appropriate vessel in the freezer (−20°C) for further use.

##### Preparation of PCL HIPEs

4PCLMA (0.4 g) and the surfactant Hypermer (10% w/w of polymer) were added into a glass vial and heated to 40°C to dissolve surfactant. Solvent blend [150% w/w of polymer, 80% chloroform, 20% toluene (w/w)] and PI (10% w/w of polymer) were added in 4PCLMA-surfactant mixture, respectively, and mixed at 375 rpm using a magnetic stirrer for 1 min at RT. Once the homogeneous mixture formed, 2.5 mL of water (internal phase volume 80% v/v) was added dropwise in 3 min and the emulsion was mixed further 30 s more.

##### The polymerization of PCL HIPEs

**Figure 1:**
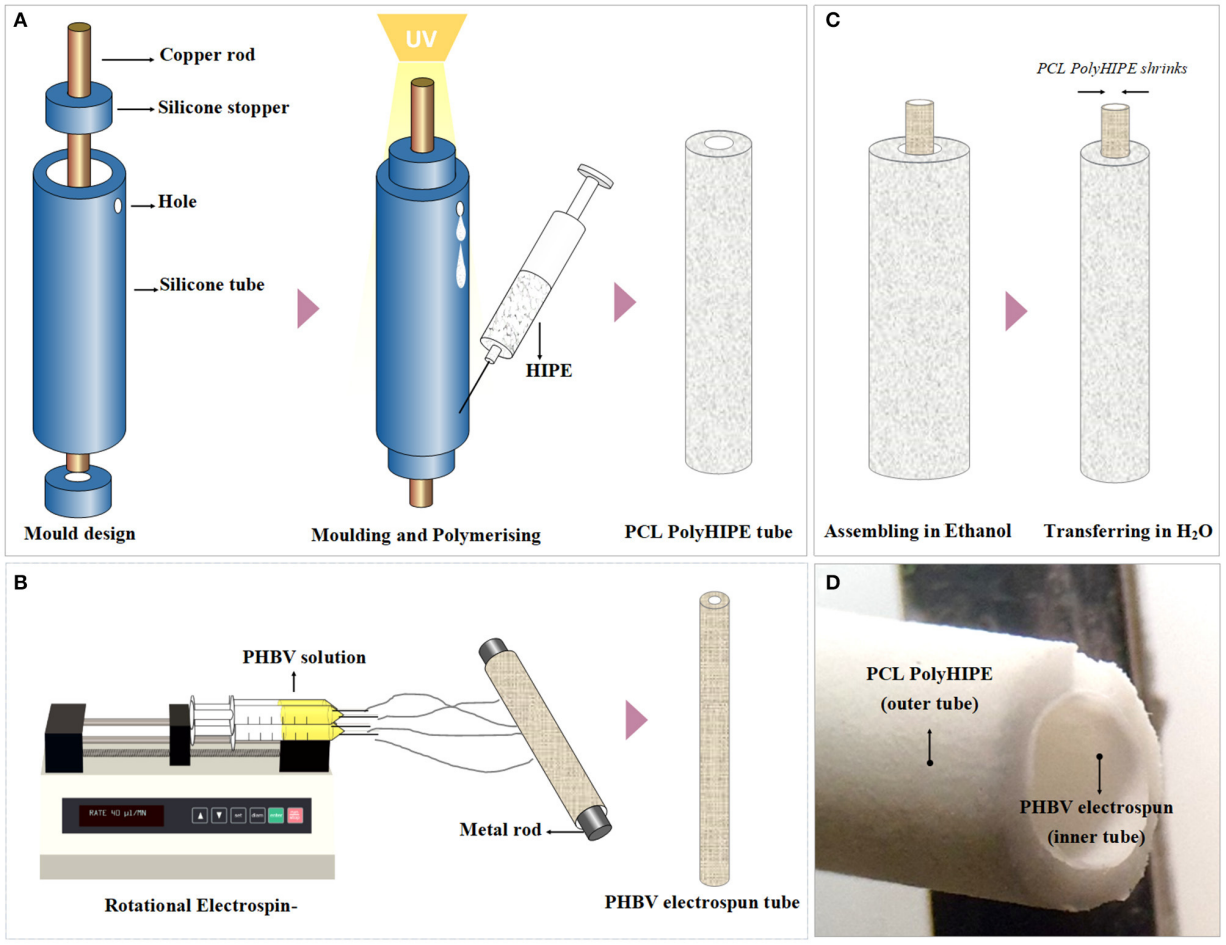
Manufacturing route of the polymeric bilayer tubes **(A)** Developed molding system to manufacture PCL PolyHIPE tubes, **(B)** electrospinning setup for manufacturing of PHBV electrospun tubes, **(C)** assembling PCL PolyHIPE and PHBV electrospun tubes to create bilayer tubes, and **(D)** macroscopic view of the bilayer tube.

A mold made of silicone tubes, stoppers, and a copper rod was assembled (Figure 1A). PCL HIPE was injected into the mold by using a syringe and polymerised for 3 min on both sides using a UV curer with a 100 W.cm^−2^ UV bulb (Omnicure Series 1000, Lumen Dynamics, Canada).

The resulting parts were recovered, soaked in 100% methanol for 24 h with four changes to remove any remaining contaminants of surfactant, solvent or uncured material. Then the samples were left in methanol [50% (v/v) in water] for 24 h and then in water for a further 24 h. Finally, the samples were taken out from the water and left in the freezer (−80°C) for an hour then transferred into a vacuum oven and left for a day to preserve the porous structure of PCL PolyHIPE without any collapse.

#### Manufacturing of the Inner Tube by Electrospinning

Poly3-hydroxybutyrate-co-3-hydroxyvalerate (PHBV) [10% (w/w)] pellets were dissolved in DCM:methanol (90:10 w/w) solvent blend, and the solution (~5 mL) was loaded into 5 mL syringes fitted with 0.6 mm inner diameter blunt syringe tips. The syringe was then placed in a syringe pump (GenieTMPlus, KentScientific, Connecticut, USA). L-Shape T9 Torx key (~2.5 mm diameter) (purchased from a local supplier) was used as the mandrel and placed at a distance of 17 cm from the needle tip (Figure 1B). The rotator and the pump were set to 250 rpm and 40 μL/min, respectively. A 17-kV voltage was applied to both the collector and the tips. The solution was then electrospun at RT for 10 min.

#### Air Plasma Treatment to Improve Cell Attachment

After fabrication of the inner and outer tubes, both tubes were treated with air plasma (Diener Electronic, Ebhausen, Germany) with a power of 50 W and a pressure of 0.8 mbar for 60 s to improve cell attachment to the hydrophobic surfaces as demonstrated in our previous work (Figure 1D) (Aldemir Dikici et al., 2019a).

#### Assembling of PCL PolyHIPE and PHBV Electrospun Tubes

PCL PolyHIPE tube was soaked in ethanol, removed and the excess alcohol was shaken off. Without letting it dry, the PHBV electrospun tube was inserted into PCL PolyHIPE tube, and they were soaked in ethanol and gradually transferred into 70% ethanol for sterilization and then transferred into PBS for complete integration of two layers which occurred due to the shrinkage of PCL PolyHIPE layer. Then 80 holes per tube were pierced using 23 G syringe needles as escape holes as described previously (Dew et al., 2016) for enabling the migration of the ECs from the inner PHBV tube to the outer PCL PolyHIPE layer.

#### Scanning Electron Microscopy

Micro-architectures of PCL PolyHIPE, PHBV electrospun, and the bilayer tubes were examined using a scanning electron microscope (SEM). All samples were gold-coated with a voltage of 15 kV for 2.5 min using a gold sputter coater (Edwards sputter coater S150B, Crawley, UK) to increase conductivity. SEM (Philips/FEI XL-20 SEM; Cambridge, UK) was used with 10 kV power.

### Design and Manufacturing of the Chamber

A chamber with a lid was manufactured using 3D printing technique to serve as a reservoir for culture media and to enable the connection of the 3D bilayer tubes to lateral flow.

**Figure 2:**
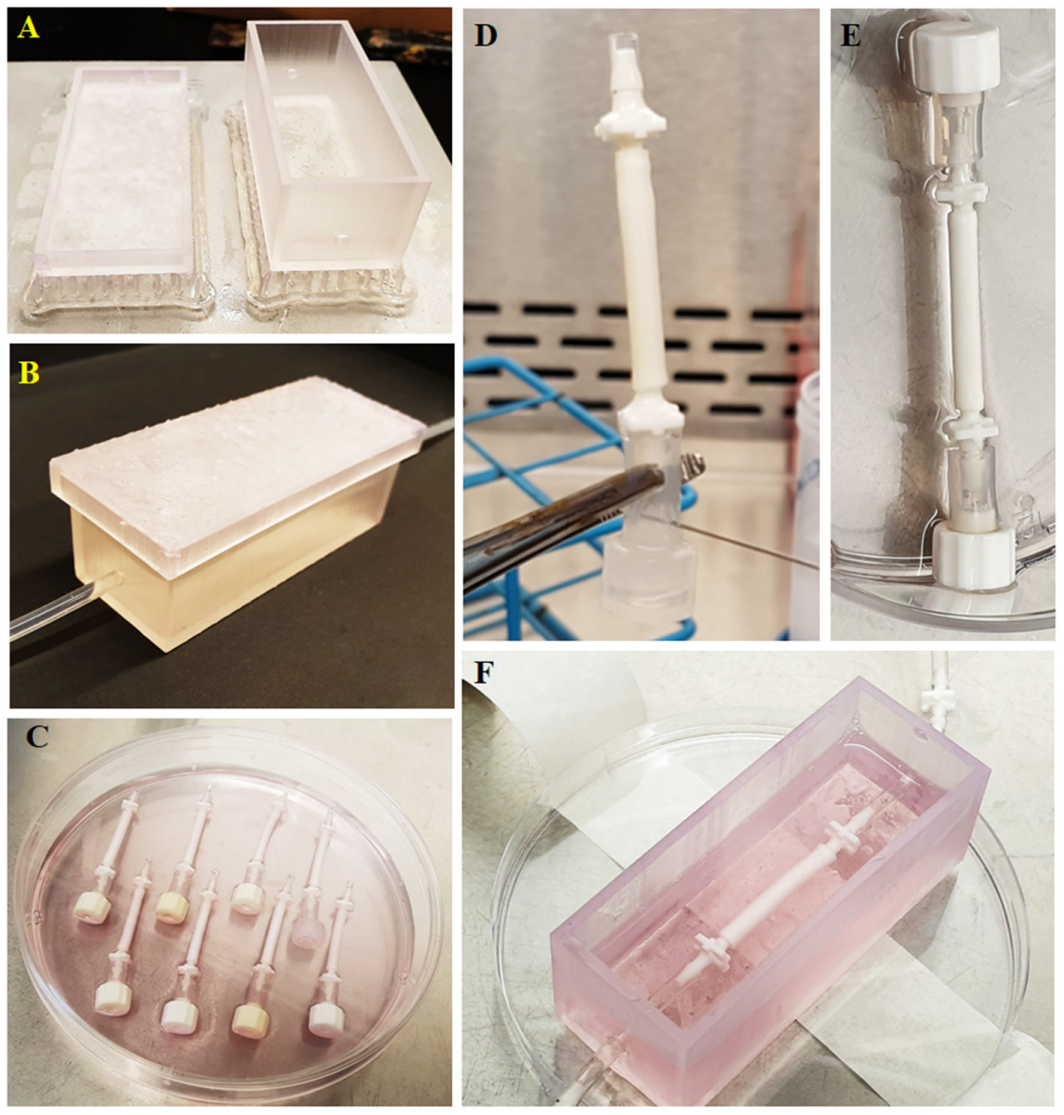
Route followed starting from the chamber manufacturing to the implementation of the tubes to the fabricated chamber. **(A)** Manufactured chamber using 3D printing, **(B)** implementation of the tubing to the chamber, **(C)** preparation of the tubes for seeding, **(D)** administration of the cell suspension into tubes using a syringe, **(E)** closing the other end with a cap to prevent leakage of the cell suspension from the tube upon seeding, and **(F)** implementation of the cellularized tube to the chamber.

The 3D models of the chamber and the lid were designed using Autodesk Inventor Professional 2020 (San Rafael, CA, USA). The chamber had inner dimensions of 70 × 25 × 25 mm with 2 mm material thickness and with input and output holes which have an inner diameter of 3 mm. The lid had 75 × 30 × 5 mm inner dimensions with 2 mm material thickness. The model was then saved as a standard tessellation language (STL) file. The STL file was imported into Formlabs Form 2 printer (Somerville, MA, USA) and printed using the resin; Dental LT Clear (Figure 2A). Following this, the chamber and lid were washed with isopropanol, post-cured at 60°C for 1 h and washed with ethanol. Then the chambers were air-dried, and silicone tubes were sealed into the input and output holes of the chambers (Figure 2B). Chamber systems were sterilized using 70% (v/v) ethanol solution (in deionised water). Briefly, they were soaked in the ethanol solution in the laminar hood and left for 2 h, air-dried in the hood for 1 h and washed with PBS three times for 2 h. The connectors and the silicone tubing were sterilized by using an autoclave, and the whole system was assembled in a laminar hood under sterile conditions using sterile forceps.

### Testing the Diffusion Pattern Between the Tube and the Chamber

The bilayer tube was connected to the chamber, and the chamber was filled with 30 mL of deionised water. Trypan blue was injected into the silicone tube to be circulated, and 10 dyn/cm^2^ flow was applied to the system. Macroscopic images of the system were captured at the end of 1, 5, and 10 min to observe the diffusion of the trypan blue. At the end of the experiment, deionised water in the chamber was collected, and the volume was measured.

### Assessment of the Angiogenic Potential of 2dDR Using a Newly Developed 3D Dynamic Model

#### Rotational Cell Seeding Into Bilayer Tubes

One end of the tubes was occluded, as shown in Figure 2C and bilayer tubes were left in 70% ethanol for 2 h and then washed with PBS three times in sterile conditions. Finally, they were conditioned with media for an hour in the incubator in 24-well plates. HAECs were trypsinized, counted, and centrifuged. The cell pellet was re-suspended in fresh media, and 10^6^ cells/300 μL were injected into tubes using a syringe, as shown in Figure 2D. Once all the cell suspension was injected, the other end was closed using a cap (Figure 2E). Tubes were transferred in a 15 mL centrifuge tube with 10 mL EC GM and placed in a cylindrical rotator turning at 1 rpm in a 37°C, 5% CO_2_ humidified incubator for 4 h for the attachment of HAECs.

#### 3D Culture of HAECs in the Newly Developed Dynamic Model for the Assessment of Angiogenesis

Following cell seeding, caps in both ends of the bilayer tubes were removed, and bilayer tubes were connected to silicone tubing in the printed chamber using sterile forceps (Figure 2F). HAECs in bilayer tubes were cultured for a week. In 3D experiments, the individual effect of flow, pro-angiogenic agents and their combined effect on EC infiltration were investigated. Flow experimental groups were static (control), 1, 2, and 10 dyn/cm^2^. Then, drug experimental groups; 100 μM 2dDR and VEGF were investigated under static conditions. Finally, 100 μM 2dDR and VEGF were further investigated under 2 dyn/cm^2^ flow and compared with the static conditions.

#### Hematoxylin and Eosin (H&E) Staining

Bilayer tubes cultured with HAECs for a week were stained with H&E using a standard protocol (Fischer et al., 2008). Briefly, samples were washed with PBS and fixed with 3.7% FA. They were washed with PBS, and excess water was removed using filter paper. Meanwhile, cryomoulds were filled with OCT-TFM. Samples were embedded into it, and the rest of the volume was then filled with OCT-TFM to the top. Cryomoulds were placed into −80°C freezer and incubated for 30 min until solidified. Frozen blocks were fixed on mounting platforms, and placed into a cryostat (Leica CM1860 UV, Milton Keynes, UK) before sections were sliced at 8 μm and immediately mounted onto the surface of Thermo SuperFrost® Plus slides. For H&E staining, slides were stained with hematoxylin for 90 s and eosin for 5 min and they were dehydrated, cleared and mounted the slide using the permanent mounting medium.

#### Fluorescent Staining

Bilayer tubes were fixed with 4% PFA for 30 min and washed gently with PBS prior to submerging into 0.1% (v/v) Triton X 100 (in PBS) solution for 20 min and stained as described in section Fluorescent Staining of Cell Nuclei and Cytoskeleton. Then, the stained tubes were sectioned, as explained in section Hematoxylin and Eosin (H&E) Staining and imaged under a fluorescent microscope (Olympus IX3, Tokyo, Japan).

### Statistical Analysis

Statistical analysis was carried out using one-way and two-way analysis of variance (ANOVA) using statistical analysis software (GraphPad Prism, CA, USA). Where relevant, *n* values are given in figure captions. Error bars indicate standard deviations in the graphs unless otherwise stated.

## Results

### Evaluation of the Angiogenic Potential of 2dDR Using Established *in vitro* Assays

#### 2dDR Improves the Metabolic Activity and Proliferation of HAECs in a Dose-Dependent Manner

**Figure 3:**
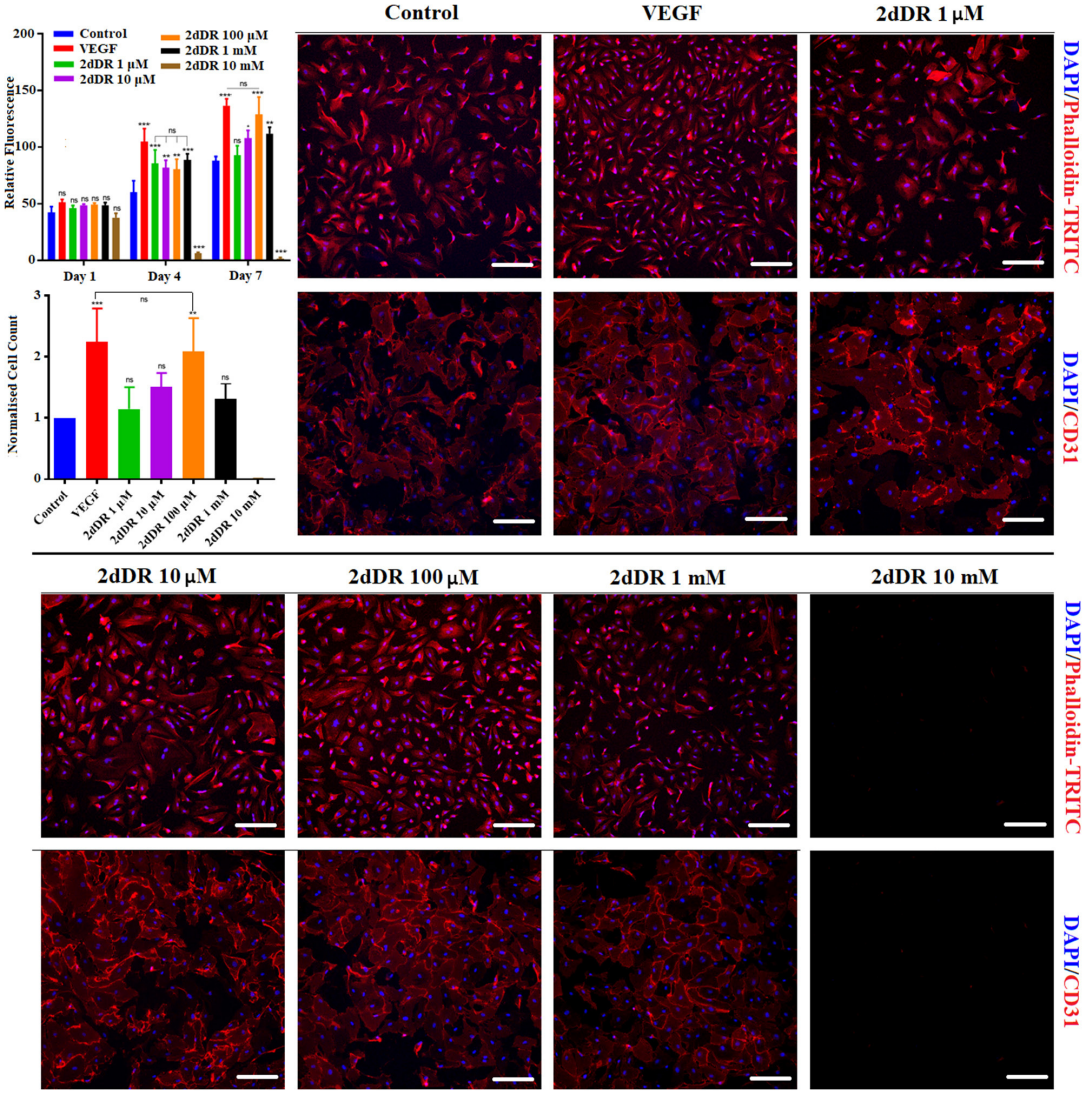
(Top graph) The metabolic activities of HAECs over 7 days and (bottom graph) the normalized number of HAECs at the end of day 7. DAPI/Phalloidin staining and CD31 expressions of HAECs at the end of day 7 when treated with different concentrations of 2dDR in comparison with VEGF and controls [***p ≤ 0.001, **p ≤ 0.01, *p ≤ 0.05, not significant (ns) *p* ≥ 0.05, *n* = 3]. Scale bars represent 200 μm.

The results of metabolic activity assays showed that 80 ng/ml of VEGF increased metabolic activities and the total number of cells as expected. The effect of VEGF was evident even at day 1 (but not statistically significant) but clearly significant by days 4 and 7. By day 7, cell number essentially doubled by the addition of VEGF (Figure 3). At 100 μM, 2dDR was as effective as VEGF in stimulating metabolic activity and cell number without any adverse effect on the appearance of cells. Lower concentrations of 2dDR were found less effective. At higher concentrations, 1 mM 2dDR was slightly less effective when compared with 100 μM, and at 10 mM concentration cells detached from the culture wells by day 4.

The results of the fluorescent staining at day 7 showed that cell proliferation was correlated with the metabolic activities of HAECs. The quantification of the fluorescent images demonstrated that when cells were treated with VEGF for 7 days, the number of cells was increased approximately 2.2-fold in comparison with the controls. Similarly, 100 μM 2dDR almost doubled the cell number at the end of day 7. No statistical difference was observed for other concentrations of 2dDR apart from 10 mM. HAECs detached from the surface of the well plate when cells were treated with 10 mM of 2dDR for 4 or 7 days.

The treatment of HAECs with VEGF and 2dDR did not affect CD31 expression of HAECs. All groups apart from 10 mM of 2dDR showed similar levels of expression of CD31, which were correlated with the number of cells at the end of day 7. No expression of CD31 was observed for 10 mM 2dDR groups, as all cells had detached. Accordingly, for the following migration experiments, 1 μM and 10 mM 2dDR concentrations were excluded as 1 μM was too low to be effective and 10 mM detached cells after exposure for several days.

#### 2dDR Enhances the Chemotactic Migration of HAECs

**Figure 4:**
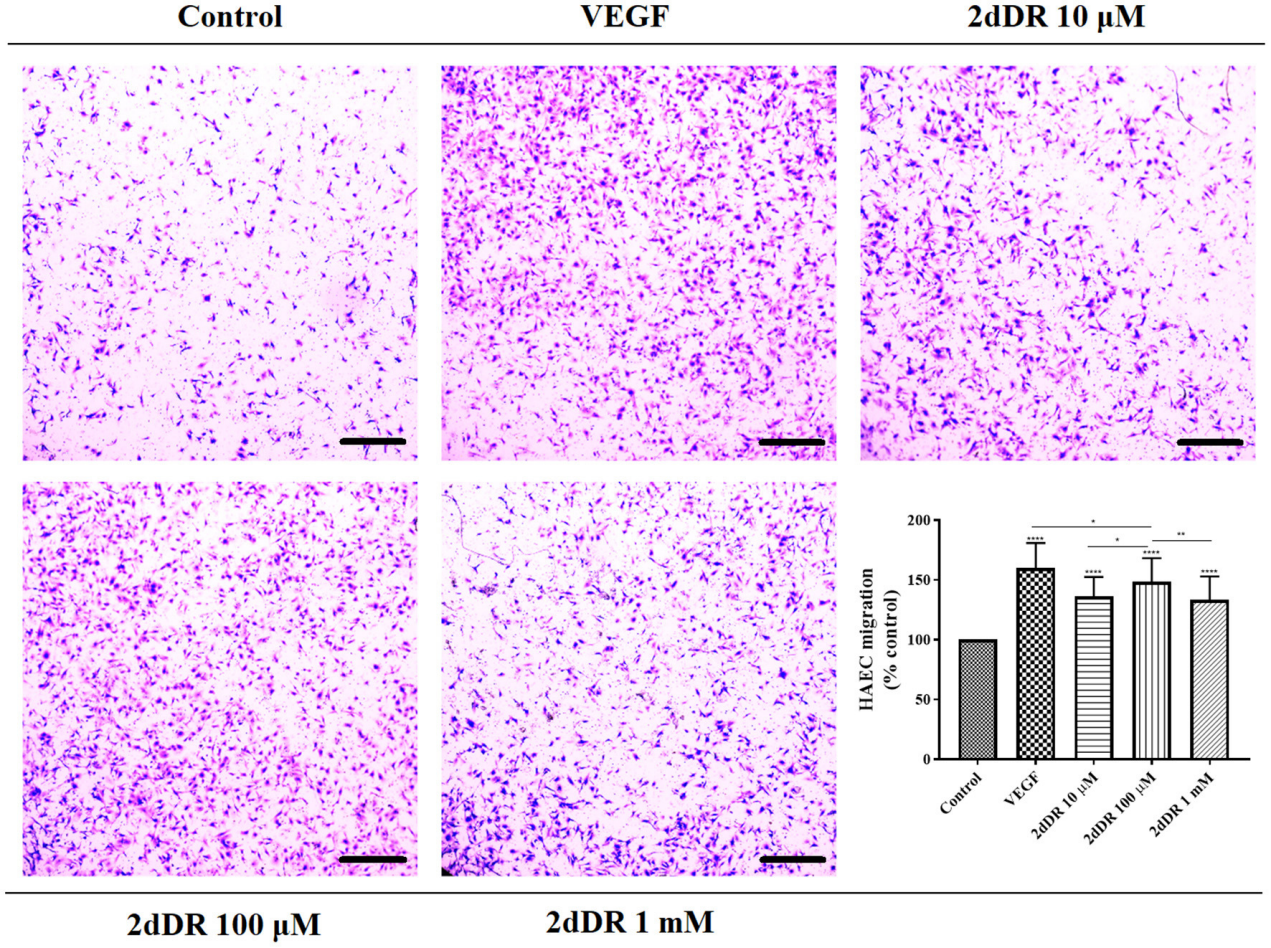
The migratory effect of different concentrations of 2dDR in comparison with VEGF and controls was evaluated by using a modified Boyden chamber assay. The quantified results were given in the graph bottom-right [****p ≤ 0.0001, **p ≤ 0.01, *p ≤ 0.05, not significant (ns) *p* ≥ 0.05, *n* = 3]. Scale bars represent 250 μm.

All three concentrations of 2dDR (10, 100 μM, and 1 mM) and VEGF significantly enhanced the chemotactic migrations compared with the control group (Figure 4). Quantification of the results indicated that the increase in the number of migrated cells was significantly different for 100 μM in comparison with the other two concentrations of 2dDR. The addition of VEGF significantly increased the number of migrated HAECs when compared with 100 μM 2dDR, which was also selected for further tube formation experiments.

#### 2dDR Improves the Ability of HAECs to Form Tube-Like Structures

**Figure 5:**
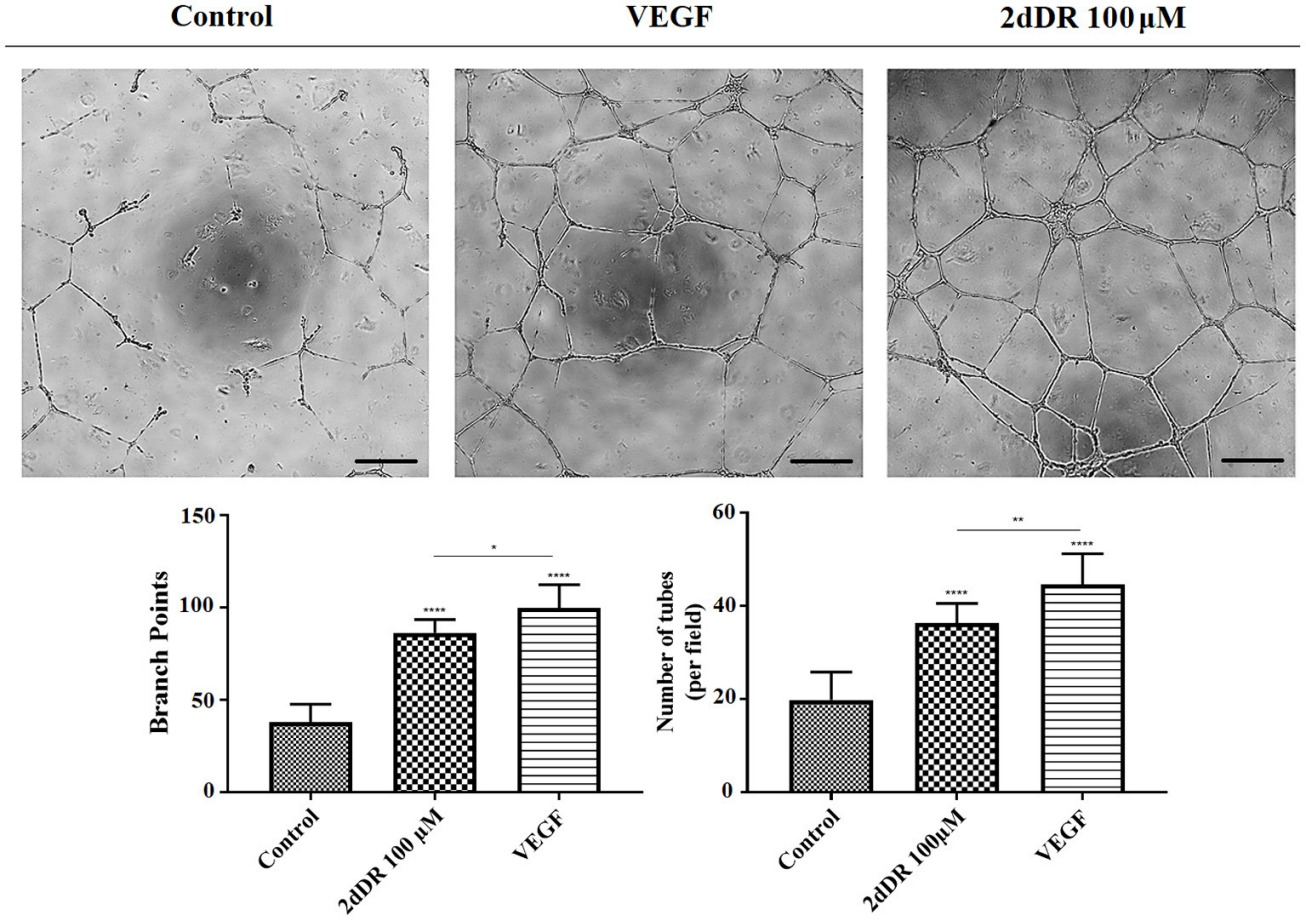
The effect of 100 μM 2dDR and VEGF on tube formation was assessed with Matrigel® tube formation assay. The quantified results of the average number of branch points and the number of tubes per field were given in the graphs given [****p ≤ 0.0001, **p ≤ 0.01, *p ≤ 0.05, not significant (ns) *p* ≥ 0.05, *n* = 3]. Scale bars represent 250 μm.

The administration of 2dDR and VEGF significantly improved the ability of HEACs to form tubes in Matrigel®, whereas cells were partly capable of forming these structures in the control group (Figure 5). The results indicated that the inclusion of 2dDR and VEGF significantly increased the number of branch points to 86 ± 7 and 100 ± 13, respectively from controls (average number of branch points: 38 ± 10). Similarly, the number of tubes per field was increased 1.8- and 2.3-fold with the administration of 2dDR and VEGF, respectively, when compared with control.

### Design, Manufacturing, and Characterization of the Developed 3D Dynamic System

**Figure 6:**
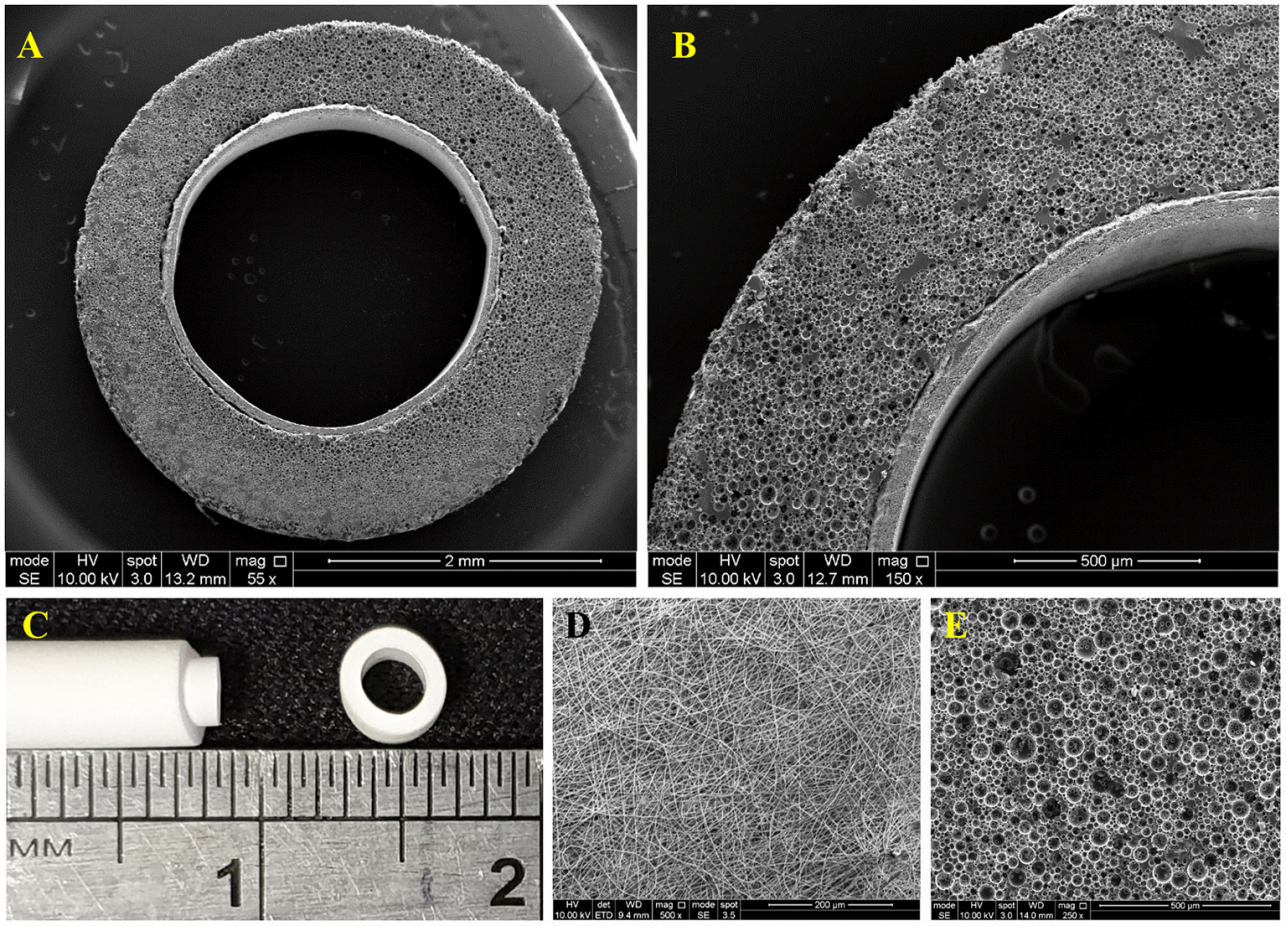
**(A,B)** SEM image of the cross-section of the bilayer tube, **(C)** Macroscopic image of the bilayer tube, **(D)** SEM images of the of PHBV electrospun, and **(E)** PCL PolyHIPE.

The 3D bilayer tubes, Figures 6A–C, which consists of an inner PHBV electrospun tube for ECs to attach and form an endothelial monolayer and an outer PCL PolyHIPE tube which provides a highly porous and interconnected environment for ECs to infiltrate was developed successfully following the manufacturing route illustrated in Figure 1.

The surface morphology of PHBV electrospun was beadless with random (non-aligned fibers) fiber orientation where the average fibers and pore sizes were 0.70 ± 0.25 and 3.64 ± 2.16 μm, respectively (Figure 6D). The outer layer, PCL PolyHIPE, had an average pore size of 30 ± 13 μm (Figure 6E).

The 3D culture chamber manufactured using 3D printing was capable of supporting the culture of HAEC in bilayer tube for periods of 7 days. There was no leakage or contamination throughout the experiments. The resin, Dental LT Clear, used for manufacturing of the chamber was reported to be biocompatible by the manufacturer. It was resilient to 70% ethanol for sterilization of the chamber. Its transparency was advantageous for bioreactor chambers to be able to allow the visual inspection of the media color without opening the lid, which reduces the risk of contamination throughout the culture. This petri-dish inspired design is reproducible, cost-effective ($15/chamber) and allows gas exchange. Its small dimensions enable multiple chambers to be used in the same incubator at the same time.

### Assessment of the Angiogenic Potential of 2dDR Using Newly Developed 3D Dynamic Model

#### Flow Mediates Proliferation and Outgrowth of ECs: Low Shear Stress Promotes Proliferation and Migration of HAECs

**Figure 7:**
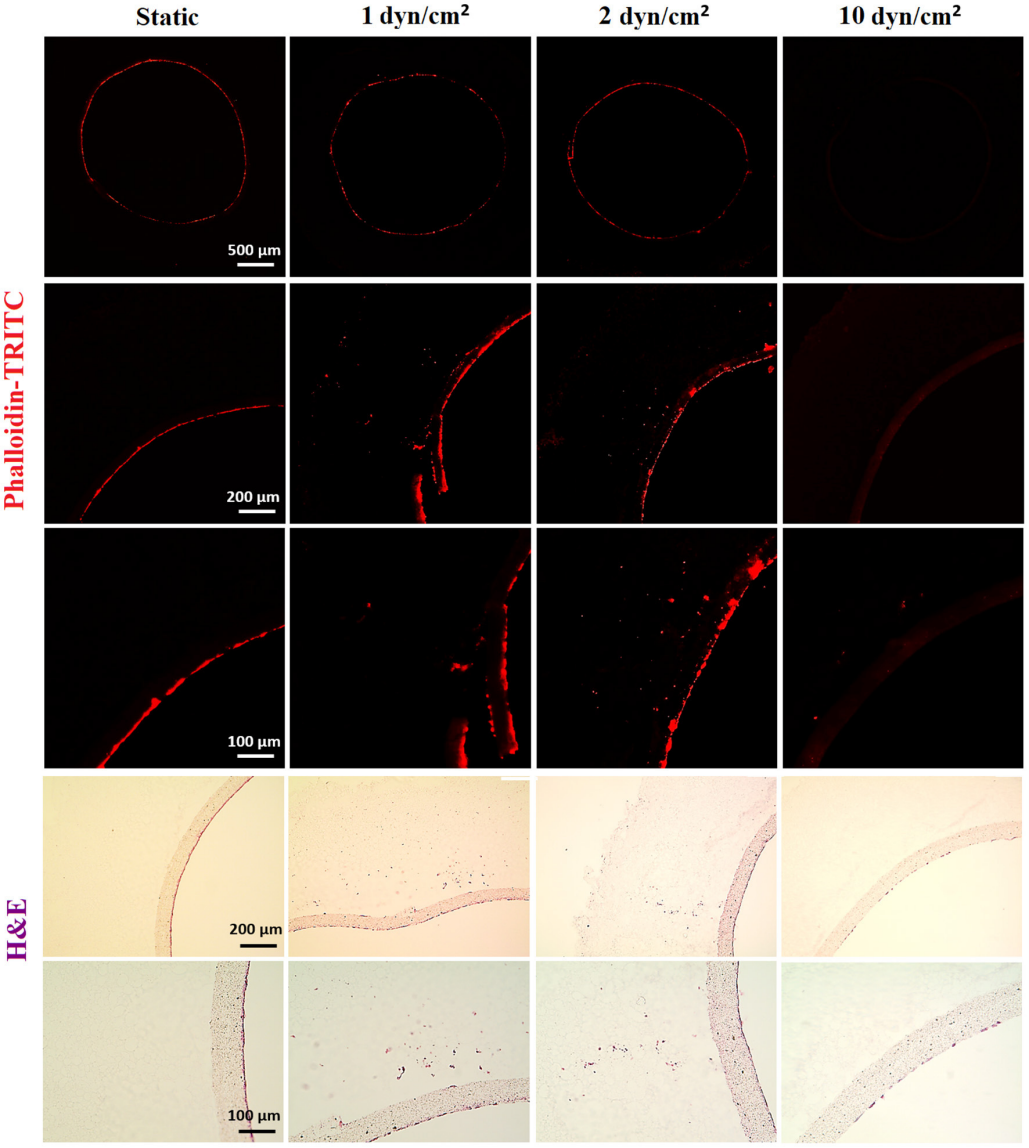
Effect of flow on the outgrowth distance, cell density and the cell monolayer formation of HAECs. (Top) Phalloidin TRITC, (Bottom) H&E staining of the sections of the bilayer tubes cultured with HAECs for a week under static culture and dynamic culture with 1, 2, and 10 dyn/cm^2^ flow from left to right, respectively.

HAECs were seeded in bilayer tubes and cultured under static, 1 dyn/cm^2^ flow, and 2dyn/cm^2^ flow formed a continuous endothelial monolayer at the end of a 1-week culture period (Figure 7). The cells cultured in tubes under 10 dyn/cm^2^ flow showed relatively poor cell distribution, which is likely due to cells were washed out because of the comparably high flow rate which gave the lowest cell density observed amongst all of the groups. However, migration of HAECs through electrospun fibers was higher under 10 dyn/cm^2^ shear stress (202.6 ± 107.7 μm) compared with the static group where no outgrowth was observed. HAEC outgrowth and cell density in tubes cultured under 2 dyn/cm^2^ flow were significantly higher and statistically different than other groups. HAECs migrated approximately 533 ± 90.8 μm in 7 days under 2 dyn/cm^2^ shear stress while the outgrowth was 231.5 ± 70.1 μm when 1 dyn/cm^2^ shear applied. Moreover, at day 7, the normalized density of cells in the tubes was significantly increased under 2 dyn/cm^2^, and it was approximately 2-, 1.4-, and 12-fold higher in comparison with static, 1, and 10 dyn/cm^2^ shear stress conditions, respectively. Fluorescent and H&E staining of the HAECs under static and flow conditions are given in Figure 7.

#### Testing the Diffusion Pattern Between the Tube and the Chamber

**Figure 8:**
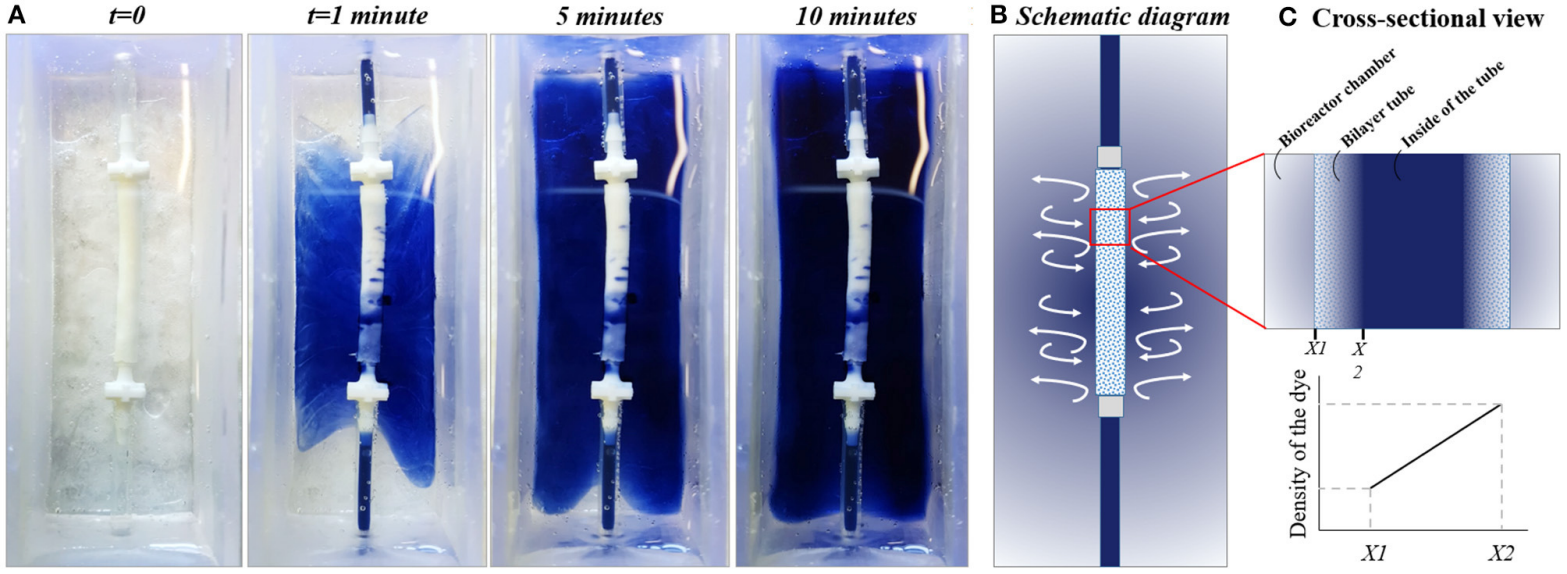
Testing the diffusion pattern between the chamber and the bilayer tube. **(A)** The diffusion of trypan blue dye from flow system to the outer chamber, **(B)** Schematic diagram of the system, and **(C)** cross-sectional view of the tube showing the dye gradient between bioreactor chamber and inside of the tube at any point till balance point.

To test the diffusion behavior between the chamber and the tube visually, the trypan blue dye was circulated in the system, and macroscopic images of the chamber were captured at 1, 5, and 10 min (Figure 8A). Images showed the gradual release of the dye from the inner tube to the chamber. Also, the volume of the liquid in the chamber was measured both at the beginning of the experiment and at the end of 10 min of circulation, and it was 30 mL at both time points.

#### 2dDR and VEGF Promotes Proliferation and Outgrowth of HAECs Under Static Conditions

In 2dDR and VEGF supplemented groups, HAECs in the electrospun tube formed a dense-packed endothelial monolayer inside the tube. No HAEC infiltration was observed in the control group which was not supplemented with any drug under static conditions whereas in 2dDR and VEGF supplemented groups, HAECs were able to migrate through PCL PolyHIPE layer approximately 182.2 ± 81.6 and 264.9 ± 88.1 μm, respectively. There was no statistically significant difference between VEGF and 2dDR supplemented tubes under static conditions in terms of HAEC outgrowth. The cellular densities in the tubes under static conditions were increased 1.2- and 1.5-fold by the addition of 2dDR and VEGF, respectively.

#### Pro-angiogenic Agents (2dDR and VEGF) and Fluid Forces Cooperate to Improve the Outgrowth and Proliferation of HAECs

**Figure 9:**
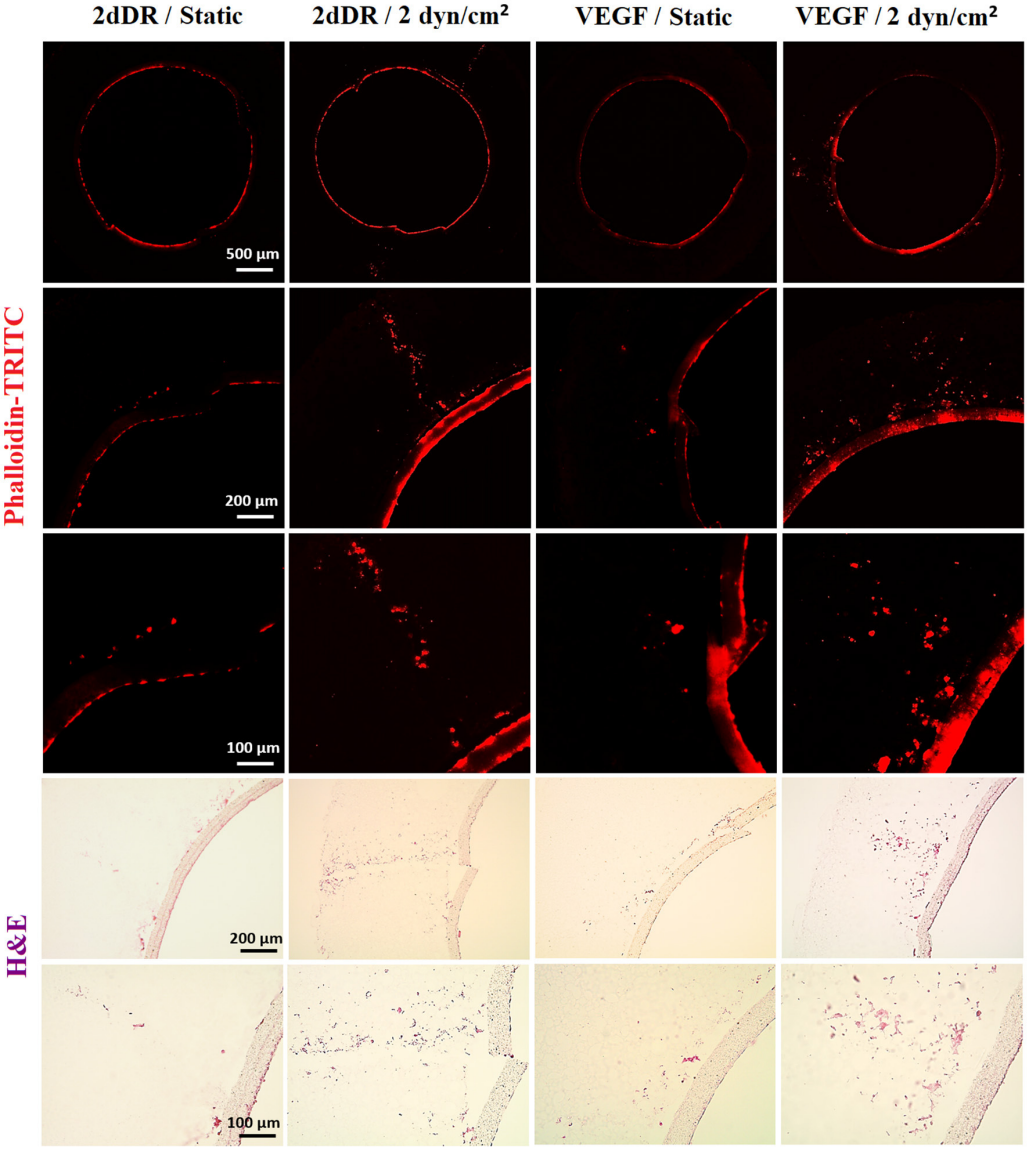
The combined effect of 2dDR, VEGF, and flow (2 dyn/cm^2^) on the outgrowth distance, cell density and the cell monolayer formation of HAECs. (Top) Phalloidin TRITC, (Bottom) H&E staining of the sections of the bilayer tubes cultured with HAECs under static culture and dynamic culture with 2 dyn/cm^2^ flow and with the implementation of the angiogenic agents (VEGF and 2dDR).

**Figure 10:**
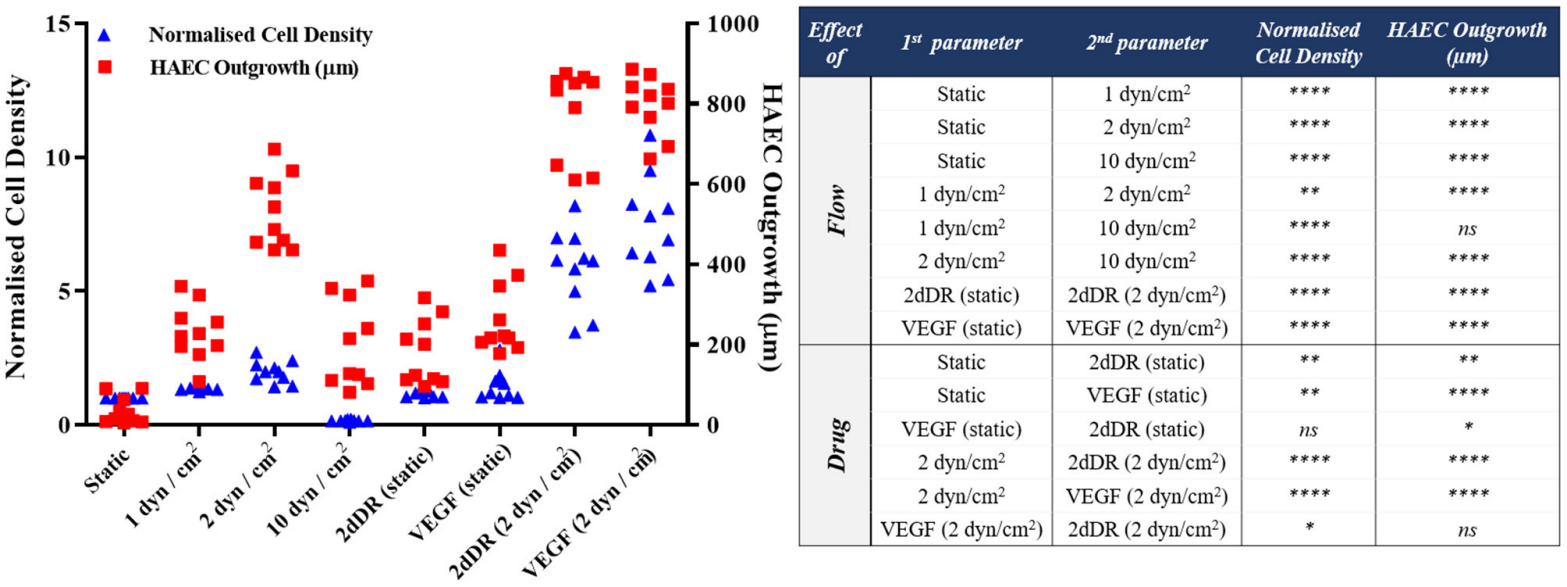
Quantification of the outgrowth distance (μm) and the cell density (normalized to the static alone group) of HAECs to show the effect of flow and drugs either in isolation or combined [****p ≤ 0.0001, **p ≤ 0.01, *p ≤ 0.05, not significant (ns) *p* ≥ 0.05, *n* = 3].

The highest distance of outgrowth and a dramatic increase in the cellular density was observed when HAECs were exposed to the simultaneous application of the angiogenic drugs and the flow. HAECs cultured under 2 dyn/cm^2^ shear stress with a growth medium supplemented with either 2dDR or VEGF migrated up to 779.8 ± 110.3 and 797.3 ± 72.5 μm, respectively. Furthermore, the cellular densities in the tubes were increased 3.8- and 3.2-fold, respectively, for 2dDR and VEGF groups under 2 dyn/cm^2^ shear stress when compared with the drug including static controls. There was no statistically significant difference observed between 2dDR and VEGF groups under 2 dyn/cm^2^ shear stress in terms of HAEC outgrowth. Fluorescent and H&E staining of the HAECs under static and flow conditions either administered with drugs or non-treated are given in Figure 9. The graph showing the quantified results of flow and drug experiments and the statistical analysis is given in Figure 10.

## Discussion

VEGF is a crucial angiogenic factor that plays a key role in promoting angiogenesis (Hoeben et al., 2004). ECs are sensitive to VEGF signaling, which regulates their proliferation, migration and survival (Olsson et al., 2006; Wang et al., 2008). Although VEGF is commonly accepted as the gold standard for promoting angiogenesis, the use of exogenous VEGF can promote the formation of leaky (Yancopoulos et al., 2000), permeable (Cao et al., 2004), and haemorrhagic (Cheng et al., 1997) vessels when administered in an uncontrolled manner. VEGF is the most widely studied pro-angiogenic factor, and several reports have established the signaling pathway responsible for its angiogenic activity (Hoeben et al., 2004; Koch et al., 2011).

2dDR is a promising pro-angiogenic alternative which has been reported to have chemotactic and angiogenic activity by our group and other researchers using a range of current angiogenic assays: Boyden chamber assay (Uchimiya et al., 2002), tube formation assay (Uchimiya et al., 2002), CAM assay (Haraguchi et al., 1994; Dikici et al., 2019b), and rat wound healing models (Yar et al., 2017; Azam et al., 2019). However, the dose-dependent response to 2dDR still remains to be investigated *in vitro* to determine what is the effective concentration range to drive angiogenesis at the cellular level.

**Figure 11:**
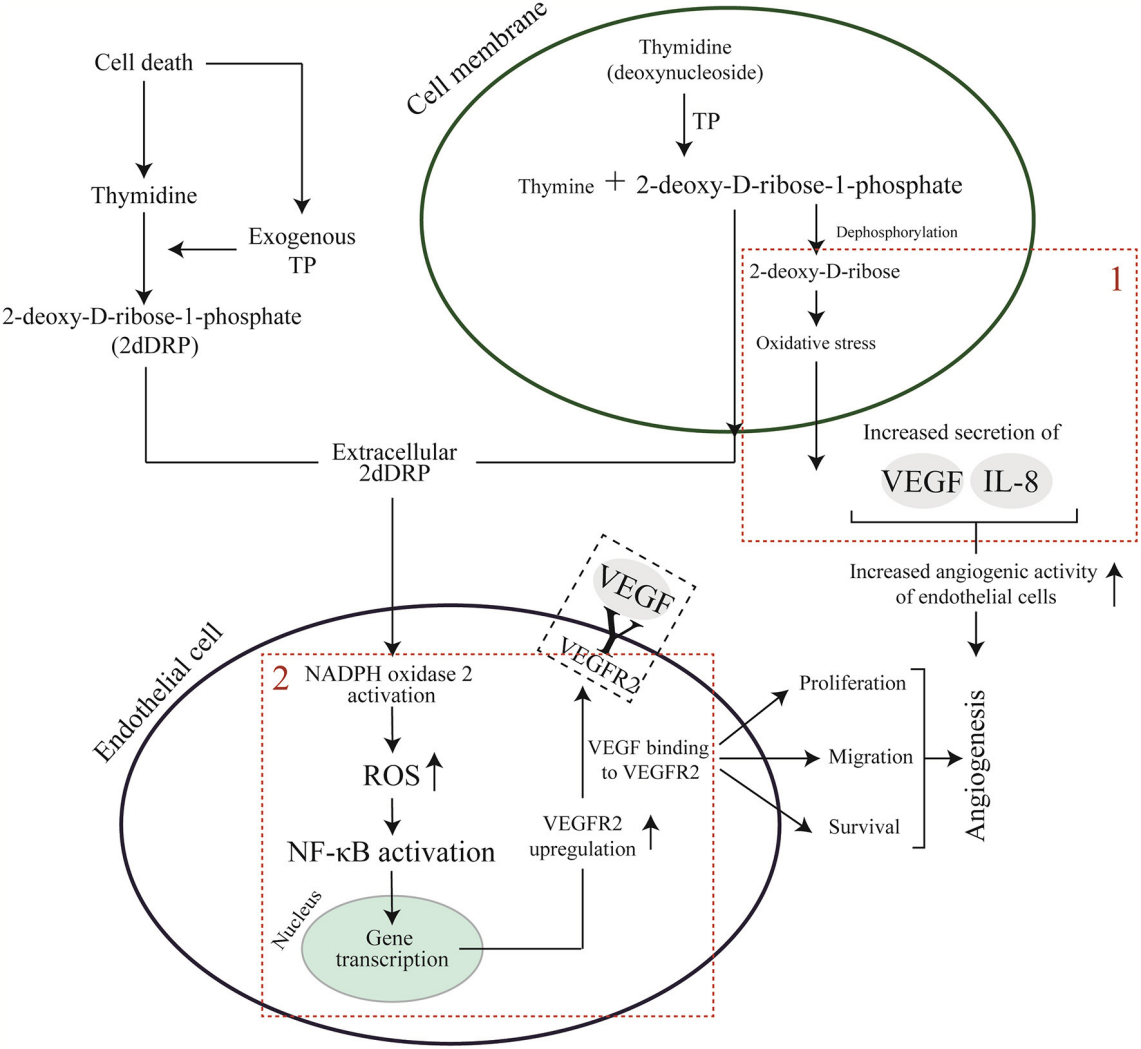
Proposed pathways for VEGF and 2dDR to promote angiogenesis. (1) endogenous generation of 2dDR stimulates the production of angiogenic factors via stimulation of oxidative stress. (2) 2dDRP takes a role in the upregulation of VEGFR2 via generation of ROS and NF-κB.

In contrast to VEGF, the mechanism of action of 2dDR remains still unclear. Only a few studies have proposed a pathway to explain the angiogenic mechanism behind this small sugar molecule. Briefly, two main mechanisms have been proposed when it comes to the angiogenic activity of 2dDR at the molecular level. In the first proposed mechanism, researchers suggest the endogenous production of 2dDR by enzymatic degradation of thymidine to thymine promotes oxidative stress and consecutively stimulates the secretion of angiogenic factors such as VEGF and interleukin-8 (IL-8) which can be internalized by ECs and promote angiogenesis (Brown et al., 2000; Sengupta et al., 2003; Nakajima et al., 2012). In the second mechanism, which has been recently studied by Vara et al. (2018), the 2dDR-1-phosphate (2dDRP) is produced either internally by cells which express TP such as macrophages, platelets and cancer cells and then secrete this extracellularly, or TP may be released from injured cells to act on enzymatic degradation of thymidine and generation of 2dDR extracellularly. 2dDRP then can be taken up by ECs, and this then activates NADPH oxidase 2 (NOX2) which later acts on nuclear factor kappa B (NF-κB). NF-κB then upregulates the VEGF receptor 2 (VEGFR2) and thus drives VEGF-dependent angiogenesis. Both mechanisms are summarized in Figure 11.

As must be evident in these early days of studying 2dDR, the clarification of the mechanism of action of 2dDR remains to be definitely established, and this is beyond the remit of this study. In this study, we wished to learn more about the biological activity of this sugar using a range of well-established *in vitro* models and in particular to establish the effective concentration range of this small sugar.

To test the angiogenic activity of added pro-angiogenic factors, biomechanical stimulus and cells, angiogenesis models are important tools (Staton et al., 2009), and the current angiogenesis assays can be divided into three categories: *in vitro* assays which focus on evaluating proliferation, migration, and tube formation capabilities of ECs (Hayakawa et al., 2015), *ex-vivo* assays and *in vivo* assays (Stryker et al., 2019). Although *in vivo*, assays are the most representative and reliable models for the evaluation of angiogenesis, they are also expensive, technically difficult, time-consuming and ethically questionable. On the other hand, *in vitro* angiogenesis assays are inexpensive, quick, technically simple, and reproducible, but they are usually based on evaluating only one aspect of angiogenesis, and as such do not represent the complexity of angiogenesis which occurs *in vivo*. A comparison of current angiogenesis assays in terms of cost-effectivity, ethical concerns, reproducibility, the requirement of special skills, representations of the physiological angiogenesis and the duration of the assay are given in Table 1.

**Table 1:** Comparison of current *in vitro, ex vivo*, and *in vivo* angiogenesis assays with the developed dynamic 3D angiogenesis model (– = absence, + = very low, ++ = low, +++ = low/medium, ++++ = medium, +++++ = medium/high, ++++++ = high).

	**Models**	**Cost**	**Ethical concern**	**Reproducibility**	**Special skill requirement**	**Representation of the physiological angiogenesis**	**Duration of the assay**	**References**
*In vitro*	Proliferation assays	+	–	++++++	+	+	++/+++	Arbiser et al., 1998; Goodwin, 2007; Logie et al., 2010; Chiu et al., 2012
	Migration assays	++	–	++++++	+	+	+	Kye et al., 2004; Logie et al., 2010; Chiu et al., 2012
	Tube formation assays	++	–	++++	++	+	+	Langenfeld and Langenfeld, 2004; Hung et al., 2007; DeCicco-Skinner et al., 2014
	3D dynamic angiogenesis model	+	–	++++++	+	++/+++	++	*This study*
*Ex vivo*	Aortic ring/arch assays	++	++	+++	+++	+++	+++	Masson et al., 2002; Auerbach and Muthukkaruppan, 2012; Baker et al., 2012
	Retinal assay	++	++	+++	+++	+++	++	Sawamiphak et al., 2010; Rezzola et al., 2013
*In vivo*	*In-ovo* CAM assay	++	+++	+++	++++	++++	++++	Vargas et al., 2007; Lokman et al., 2012
	*Ex-ovo* CAM assay	++	+++	+++	+++++	++++	++++	Aldemir Dikici et al., 2019a; Dikici et al., 2019a,b; Mangir et al., 2019
	Dorsal skinfold chamber assay	++++++	++++++	++	++++++	++++++	+++	Rücker et al., 2006; Laschke et al., 2011
	Sponge/Matrigel® plug assay	++++++	++++++	++	++++++	++++++	++++	Akhtar et al., 2002; Malinda, 2009
	Corneal assay	+++++	++++++	++	+++++	+++++	+++	Ziche et al., 1994; Arbiser et al., 1998
	Zebrafish assay	++	+++	++	++++	++++	++	Serbedzija et al., 1999; Nicoli and Presta, 2007

When all the drawbacks of these methods are considered, there is a need for a 3D dynamic system which enables the study of angiogenesis *in vitro* in a more physiological complex 3D environment which includes the introduction of flow which is a major stimulus for neovascularization *in vivo*.

Experiments in this study were designed with three key objectives; (i) to assess the angiogenic activity of 2dDR using established 2D *in vitro* angiogenesis assays, (ii) to develop a 3D dynamic system to be used for testing of pro-angiogenic agents, (iii) to evaluate the efficiency of 2dDR in encouraging proliferation and infiltration of HAEC using *in vitro* 3D dynamic system to study the suitability of our model to be used as a 3D *in vitro* angiogenesis model. Throughout these experiments, we used VEGF as a comparator so that we can answer how biologically effective 2dDR is compared to this well-documented major pro-angiogenic factor.

First, we have investigated the effect of 2dDR on proliferation, chemotactic migration and tube formation ability of HAECs. The results of the metabolic activity assay showed a dose-dependent response in the metabolic activity of HAECs. It was not effective when used at low concentrations (1 μM) but significantly improved metabolic activity and proliferation of HAECs when used at 100 μM and to a lesser extent 1 mM. It inhibited cellular proliferation at higher concentrations (10 mM) by detaching the cells. 100 μM 2dDR was found ~95% as potent as VEGF in terms of enhancing the proliferation of HAECs over 7 days. Following the metabolic activity assay, three concentrations of 2dDR (10, 100 μM, and 1 mM) were further examined for enhancing the chemotactic migration of HAECs using a modified Boyden chamber assay. All the 2dDR concentrations and VEGF showed a statistical difference when compared with controls in terms of migration of HAECs from the upper chamber to the lower one where the chemoattractant was located. Hundred micrometer group was then selected for further experiments to evaluate how the addition of 2dDR and VEGF influences the formation of capillary tube-like structures using a well-established method of assessing angiogenesis, Matrigel® tube formation assay (Ponce, 2009). The administration of 100 μM of 2dDR and 80 ng/ml of VEGF increased the number of capillary tubes formed and the number of branch points.

Thus, the *in vitro* assessment of the angiogenic activity of 2dDR showed it to be dose-dependent reproducing all of the actions of VEGF with 2dDR stimulating proliferation, migration and tube formation of HAECs. Hundred micrometer of 2dDR was essentially equivalent to 80 ng/mL of VEGF, and this was selected for further evaluation in the newly developed 3D dynamic angiogenesis model.

The 3D dynamic system was designed in a tubular form as seen in tissue engineering vascular graft design to enable the application of lateral flow (Nieponice et al., 2008; Kurobe et al., 2015; Ye et al., 2015). It was designed to be two-layers and manufactured by combining two different manufacturing methods, electrospinning, and emulsion templating.

The inner tube was manufactured using electrospinning, and it is made of nanofibres to serve as a suitable environment for HAECs to attach, proliferate and form a monolayer to represent an endothelium. Nanofibres have been shown to provide better surface properties for ECs to adhere to and to proliferate on than microfibers (Keun Kwon et al., 2005; Venugopal et al., 2008; Beachley and Wen, 2010). This is likely due to the nanofibers being structurally similar to the ECM of natural tissue with their submicron-scale topography and highly packed morphology (Keun Kwon et al., 2005; Gunn and Zhang, 2010). Furthermore, nanofibres made of PHBV have previously been shown to be a suitable environment for ECs to form an endothelial monolayer (Dew et al., 2016). Although nanofibres are favorable for the formation of the endothelium layer, these close-packed fibers act as a barrier to cell infiltration (Aldemir Dikici et al., 2019a).

In contrast, the outer tube was designed to serve as a suitable environment for cell infiltration. An emulsion templating method was used for the manufacturing of the outer layer as it enables fabrication of the scaffolds with high interconnectivity and up to 99% porosity. We have recently reported the manufacturing route of PCL based PolyHIPEs, and we have also shown the biocompatibility, and structural suitability of these scaffolds in terms of cell infiltration (Aldemir Dikici et al., 2019a,b).

Molding was used for the fabrication of the PolyHIPE tubes. The manufacturing route of PolyHIPE tubes using 3D laser patterning has previously been reported (Johnson et al., 2013). However, the production of PolyHIPE based 3D structures needs bespoke stereolithography set-ups and careful optimisation of the printing ink, the print speed of the stage and irradiation intensity, to produce good quality prints with PolyHIPEs. Thus, the use of molds is a practical and convenient method to fabricate PolyHIPEs with uncomplicated designs.

Following the fabrication of the individual layers separately, we combined them by taking advantage of the high swelling degree of PolyHIPEs in organic solvents (Busby et al., 2001). The outer diameter of the PHBV electrospun tube and the inner diameter of the PCL PolyHIPE tube were designed to be 2.4 mm when they were soaked in water or culture media. When the PCL PolyHIPE tube was transferred from water to ethanol, the diameter increased by more than 10% and enabled the insertion of the PHBV tube into the PolyHIPE tube. Then, the bilayer tube was transferred into the water, and a shrink-fit connection between the two layers was obtained (Figures 6A–C). After combining PHBV electrospun and PCL PolyHIPE tubes, they maintained their structural integrity, and no delamination was observed at any stage of the experiments over 7 days.

3D experiments were conducted to investigate the effect of (i) flow, (ii) angiogenic agents, and (iii) their combined effect on cellular density and the outgrowth distance of HAEC from the inner tube to PCL PolyHIPE layer. The inner diameter of the 3D tube was 2.4 mm to partially represent the diameter of big blood vessels such as arteries or veins, and accordingly, the shear rates used in this study ranged between 1 and 10 dyn/cm^2^ to mimic the physiological shear rates observed in some arteries and veins (Wasserman and Topper, 2004; Chatterjee, 2018).

The results of the 3D flow experiments showed that static culture and lower shear stresses (1 dyn/cm^2^ and 2 dyn/cm^2^) enabled the formation of a continuous endothelial monolayer, but the application of higher stress (10 dyn/cm^2^) resulted in a discrete monolayer of HAECs. This is possibly due to the cells being washed out at the high rate of flow. Similarly, Kitagawa et al. demonstrated a rapid decrease in the number of cells attached to their tubular scaffolds due to the high flow shear stress (Kitagawa et al., 2006). Low shear stress promoted the outgrowth of HAECs for over 7 days. 2 dyn/cm^2^ shear stress significantly increased the outgrowth distance of HAECs and normalized cell density when compared with 1 and 10 dyn/cm^2^, whereas no outgrowth was observed under static culture conditions. Our findings of the effect of flow experiment on ECs are consistent with the literature (Mohan et al., 1997, 1999). ECs are mechanosensitive, and they have been reported to show phenotypic and functional changes based on various flow patterns (Chistiakov et al., 2017). Shear stress caused by laminar flow has been reported to reduce the apoptosis while increasing the VEGF expression (dela Paz et al., 2012) and demonstrated to regulate EC migration (Simmers et al., 2007). Barron et al. reported a higher EC number and infiltration under flow conditions compared to static culture (Barron et al., 2012). Similarly, Sprague et al. demonstrated that the migration of HAECs onto a prosthetic material was positively influenced by shear stress (Sprague et al., 1997). Urbich et al. showed that the application of shear stress stimulates migration of human umbilical vein ECs in a flow rate dependent manner being at least as effective as VEGF (Urbich et al., 2002). Mohan et al. reported increased angiogenesis-related nuclear factor-kB (NF-κB) activity when HAECs were exposed to low shear in comparison with high shear stress conditions (Mohan et al., 1997).

Under static conditions, administration of 2dDR and VEGF stimulated EC outgrowth and cell density when compared with the static control group (no agents administered). However, the most dramatic increase in outgrowth distance and cell density was observed when the pro-angiogenic agents (2dDR and VEGF) were administered under 2 dyn/cm^2^ shear conditions. The outgrowth distance and cell density of HAECs under flow conditions and when treated with VEGF and 2dDR were significantly higher when compared to that observed when VEGF and 2dDR were administered under static conditions. Similarly, Song et al. reported that VEGF and fluid forces cooperate to improve endothelial invasion into collagen gel matrix (Song and Munn, 2011). Studying EC outgrowth is particularly important because the formation of new blood vessels involves budding of ECs from established vasculature toward an area of hypoxia and increased the release of pro-angiogenic factors such as VEGF.

Alongside the mechanotactic stimulus, the chemotactic stimulus is the other factor that causes a migratory response of ECs (Lamalice et al., 2007). In our system, an external media reservoir was not used as the media in the chamber was sufficient to keep the cells alive for a 7-day culture period. The bilayer tubes were porous enough to enable the application of flow and the diffusion of the media between the media the inside of the tube and the media in the chamber, as shown in the time-lapse figure (Figure 8A), the trypan blue dye was spread over the chamber by this time and as there was no volume change observed in the chamber, which indicates the liquid transport both from the tube to the chamber and the chamber to the tube. But as this transportation takes time, even after 10 min trypan blue did not cover the top of the tube. During the culture of HAECs in the tubes, there was a continuous nutrient consumption in the inside of the tube by the cells, which probably created a concentration gradient between the circulating media (in the tube) and the media in the chamber. As the circulating media (represented with trypan blue, Figures 8B,C) was comparably exhausted in terms of supplemented angiogenic agents (2dDR and VEGF) compared to media in the chamber (represented with water, Figures 8B,C), the migration of HAECs from the inner surface of the electrospun tube toward outside PolyHIPE layer can be explained by the chemotactic stimulus for ECs due to the abundance of the drugs in the chamber when compared with the inner tube where perfusion occurs.

The current study makes a novel contribution to the literature on understanding the dose-dependent angiogenic activity of 2dDR *in vitro* and by demonstrating a novel 3D model which can be used to study angiogenesis *in vitro* in a 3D dynamic environment. Herein, we showed that the 3D *in vitro* model we developed gave results consistent with the established angiogenesis assays for testing of the angiogenic effect of 2dDR in comparison with VEGF. Our model enables users to monitor cell proliferation and migration simultaneously under more physiologically relevant conditions. Our bilayer tubular system will also enable the future co-culture of ECs with another cell line; for example, seeding bone cells into the PolyHIPE tube while ECs are in the electrospun tube to study angiogenesis in bone tissue engineering. This new 3D dynamic model will be suitable for studying several aspects of angiogenesis. For instance, quantitative assessment of angiogenesis markers may give a better insight into the response of ECs to an external stimulus and to flow.

## Conclusion

In the present study, we showed that 100 μM 2dDR could be used to promote HAEC proliferation, migration and tube formation *in vitro* and was almost as effective as 80 ng/mL VEGF. A 3D bilayer dynamic system which enables one to study angiogenesis *in vitro* was successfully fabricated by combining electrospinning and emulsion templating. The angiogenic activity of 2dDR was evaluated in the 3D dynamic system compared to VEGF under static and flow conditions. Both agents improved EC density in the tube and the outgrowth of cells under either static or 2 dyn/cm^2^ shear stress conditions.

The results demonstrate that 2dDR at 100 μM can essentially be used as an alternative to VEGF in every aspect of EC biology we studied including their assessment in a 3D dynamic system developed in this study which has considerable potential to be used for the assessment of the angiogenic potential of different flow regimes and pro-angiogenic agents *in vitro*.

## Data Availability Statement

The datasets generated for this study are available on request to the corresponding author.

## Author Contributions

SD and BA contributed equally to the experimental design, analysis, acquisition, interpretation of data, statistical analysis, and drafting of this paper. SB and CS provided technical knowledge, equipment training, and assistance on the revision of the manuscript. MB, EE, SM, GR, and FC contributed with their supervision and critical revision and editing of the manuscript for important intellectual content. All authors participated in revising the paper and approved the final manuscript.

### Conflict of Interest

The authors declare that the research was conducted in the absence of any commercial or financial relationships that could be construed as a potential conflict of interest.
